# Government and charity funding of cancer research: public preferences and choices

**DOI:** 10.1186/s12961-015-0027-6

**Published:** 2015-09-03

**Authors:** Koonal Kirit Shah, Jon Sussex, Karla Hernandez-Villafuerte

**Affiliations:** Office of Health Economics, Southside 7th floor, 105 Victoria Street, London, SW1E 6QT UK

**Keywords:** Cancer, Charitable giving, Crowding, Donor behaviour, Medical research, Stated preferences, Tax

## Abstract

**Background:**

It is unclear how the public would respond to changes in government decisions about how much to spend on medical research in total and specifically on major disease areas such as cancer. Our aim was to elicit the views of the general public in the United Kingdom about how a change in government spending on cancer research might affect their willingness to donate, or to hypothecate a portion of their income tax payments, to cancer research charities.

**Methods:**

A web-based stated preference survey was conducted in 2013. Respondents considered hypothetical scenarios regarding changes in the levels of government funding for medical research. In each scenario, respondents were asked to imagine that they could allocate £100 of the income tax they paid this year to one or more medical research charities. They were asked how they wished to allocate the £100 between cancer research charities and medical research charities concerned with diseases other than cancer. After having been given the opportunity to allocate £100 in this way, respondents were then asked if they would want to reduce or increase any personal out-of-pocket donations that they already make to cancer research and non-cancer medical research charities. Descriptive analyses and random effects modelling were used to examine patterns in the response data.

**Results:**

The general tendency of respondents was to act to offset hypothetical changes in government spending. When asked to imagine that the government had reduced (or increased) its spending on cancer research, the general tendency of respondents was to state that they would give a larger (or smaller) allocation of their income tax to cancer research charities, and to increase (or reduce) their personal out-of-pocket donations to cancer research charities. However, most respondents’ preferred allocation splits and changes in personal donations did not vary much from scenario to scenario. Many of the differences between scenarios were small and non-significant.

**Conclusions:**

The public’s decisions about how much to donate to cancer research or other medical research charities are not greatly affected by (hypothetical) changes to government plans about the amount of public funding of cancer or other medical research.

## Background

Investment in medical research contributes to the continuing improvement of human health and wealth. Medical research is funded from three sources: government allocation of tax funds, medical research charities disbursing donors’ funds, and private industry investing for commercial gain. There is a substantial literature on the relationship, generally determined to be one of complementarity, between government and private industry funding of medical research (much of that literature has been previously summarised [1]). There is, however, little evidence about the relationship between government and charity funding of medical research.

The United Kingdom has a large number of medical research charities, some of which have very large funds at their disposal. Overall, charity funding of medical research tops £1 billion annually^a^ [2] and compares with United Kingdom government funding of medical research, which is around £2.5 billion annually [3]. The deep economic recession that hit many economies in 2008 and the following years have led many governments, including the United Kingdom government, to undertake fundamental reviews of all public expenditure, including public funding of research. The question therefore arises whether a cut in public funding of medical research might prompt any offsetting increased donations by the public to medical research charities, or not, or whether it might even lead to the public being less willing to donate to medical research. We set out to determine whether and how government decisions to allocate tax funds to different areas of medical research affect the general public’s desire in the United Kingdom to fund those areas of medical research. Does government funding crowd out the general public’s willingness to fund a particular area of medical research or does it act to attract more funds from the general public, or does it have no effect either way? If the public were permitted to hypothecate a small part of their annual income tax payment to medical research charities (as is possible in Italy), how would they react to changed government priorities for funding medical research?

### Brief overview of literature

There are theoretical arguments supporting both “crowding out” effects, i.e. decreases in government funding of research lead to donors increasing their own contributions [4-8], and “crowding in” effects, i.e. decreases in government funding lead to donors reducing their own contributions [9,10]. The former may be driven by altruistic motivation: donors increase their own contributions as they learn about reductions in contributions by others. The latter may be driven by a type of signalling effect whereby government spending decisions act as a signal to donors who lack information about societal priorities [11]. The literature typically presents signalling models of charitable giving in the context of donors facing uncertainty regarding the quality of a beneficiary charity: government funding of a charity is seen as a signal of the quality of that particular organisation rather than of the cause the charity is supporting. Government funding of charities is less common in the United Kingdom than the United States, and our study concerns the impact on funding of the charitable cause, medical research, rather than of any particular charity organisation. However, a similar signalling concept may apply if donors face uncertainty about the comparative worthiness of competing charitable causes. Alternatively, decisions about whether and how much to donate may be driven by positive psychological consequences for the donor, often labelled “warm glow” or “joy of giving” effects [12]. A number of studies have tested the crowding out and crowding in hypotheses by examining panel data (e.g. [8,10,11,13-18]). Eckel et al. [19], Crumpler and Grossman [20], and Li et al. [21] adopted a different approach, using laboratory experiments to test the same or related hypotheses.

The majority of published studies report United States data and may not translate to other nations. With their United States focus, most of the studies examine the impact of direct government grants to charities themselves but such grant funding is uncommon in some other countries, including the United Kingdom.

There are very few studies that specifically examine funding for research, let alone science or medical research specifically. A notable exception is Diamond [16], who reports that United States donors view federal and private spending on basic research as complements and suggests that “*private funding could not be expected to replace lost federal funding of science*”. Furthermore, most of the studies focus on increases in government spending, whereas, given the economic climate since 2008, it would be more appropriate to consider the impact of cuts in real terms government spending.

We are not aware of any studies that have used stated preference methods to examine the complementarity or substitutability of government funding and private donations for medical research. Stated preference studies are increasingly being used in the field of health, particularly to examine public preferences regarding health care priority setting [22]. A stated preference approach was also used successfully in a recent study examining the effects of different forms of tax relief on charitable giving [23]. Although surveys comprising hypothetical questions have disadvantages, they are particularly useful when information about revealed preferences (i.e. data from observed behaviour) is unavailable – in this case, when seeking to understand the potential impact of a cut in government spending that has not yet occurred.

### Objectives of the study

The aim of our study was to elicit, using a stated preference method, the views of the general public in the United Kingdom about how a change in government spending on cancer research might affect people’s willingness to donate to cancer research charities and to hypothecate tax revenues to different areas of medical research. The latter idea is similar to the situation in Italy, where, since 2006, taxpayers have been offered the opportunity to donate 0.5% of the income tax they pay to non-profit organisations of their choosing [24]. We also sought to address the lack in the existing literature on charitable behaviour of studies that focus on medical research and charitable giving outside the United States.

We focused on cancer as a high profile disease area, research into which attracts substantial government and charity funding in the United Kingdom (not least by the grant funder of this research study: Cancer Research UK). Due to the identity of the funder of this study – which we declared to respondents up-front – we were concerned that the survey should not appear as a veiled request for donations. The “cinque per mille” income tax hypothecation policy current in Italy, referred to above, suggested a framework that would enable interesting questions to be asked about individuals’ preferences for their own taxes to be spent on different areas of medical research.

The stated preference survey was designed to answer the following key research questions:

1. If given the opportunity to allocate some of the income tax they pay this year to one or more medical research charities, how would people choose to distribute that amount between cancer research and other medical research charities?
2. Would being given the opportunity to allocate some of their income tax to cancer and other medical research charities lead people to change their existing personal out-of-pocket donations to those types of charities?
3. Would people’s preferred allocations of funds change if they were to learn that the government was cutting or increasing funding for cancer and/or other medical research?

## Methods

### Survey instrument

The survey began with some preliminary questions. First, respondents were presented with a list of well-known cancer research charities and were asked to indicate which of those charities, if any, they had given money to in the past year. An “Other” option was included to allow respondents to specify the name of a cancer research charity that they had given money to that was not included in the list. Respondents were then asked a similar question but about medical research charities focusing on diseases other than cancer (for example, the British Heart Foundation).

Second, respondents were asked to guess: (1) how much the United Kingdom government currently spends on medical research each year, in millions of pounds; and (2) the percentage of total United Kingdom government spending on medical research each year that is on cancer research. It was assumed that respondents would not know the answers to either of these questions, but we sought information on their best guesses to help us interpret their responses to later questions.

Third, respondents were asked to indicate whether they thought that United Kingdom government funding of medical research had been going up or down, or had remained about the same, over the last 3 years. They were then asked the same question, but focusing specifically on cancer research.

The main part of the survey comprised five hypothetical scenarios. In each scenario, respondents were asked to imagine that they had the opportunity to allocate £100 of the income tax they paid this year to one or more medical research charities. We focused on a sum of £100 as being large enough for respondents to care about how it would be spent, but small enough to be plausible (it equates to 1.8% of the average income tax payment), and to make it easy for respondents so-inclined to think in percentage terms when allocating the money. Respondents were asked how they wished to allocate the £100 between cancer research charities and medical research charities concerned with diseases other than cancer. The recipients of the allocation would be unnamed charities of the respondents’ choosing. An alternative approach would have been to ask respondents to allocate the £100 between individual, named charities. We opted against this for two reasons: first, because we wished to examine the interdependency, if any, between government and charity funding of medical research, not the status of different medical research charity organisations; and second, because the name of the charity funding the study had been made explicit, so asking respondents whether they would give to that particular charity might have led to biased responses.

After having been given the opportunity to allocate £100 in this way, respondents were then asked if they would want to reduce or increase any personal donations that they already make out of their own pocket to cancer research and non-cancer medical research charities and, if so, by how much. Respondents were only asked about changes to their out-of-pocket personal donations if they had earlier claimed to have made financial donations to medical research charities in the previous year (see above). These questions allowed us to test the extent to which having the opportunity to allocate some of their income tax to medical research charities might affect people’s willingness to donate from their own (post-tax) resources. Table 1 summarises the information provided to respondents in the five scenarios.

**Table 1:** **Summary of scenarios used in the survey**

**Scenario**	**Amount spent on medical research in the United Kingdom each year (£m)**				**Description**
	**Funding from government**		**Funding from charities**		
	**Cancer research**	**Other medical research**	**Cancer research**	**Other medical research**	
1	No information provided to respondent				Scenario included to capture respondents’ preferences in the absence of information about medical research funding levels
2	150	2,350	350	650	Realistic estimates of actual spending on medical research
3	50	2,450	350	650	Government reduces its spending on cancer research and spends that money instead on other medical research
4	50	2,350	350	650	Government reduces its spending on cancer research; spending on other medical research remains unchanged
5	250	2,250	350	650	Government increases its spending on cancer research and reduces its spending on other medical research by the same amount

In scenario 1, no information was provided about research funding levels. In scenario 2, respondents were presented with rounded estimates of how much the government and charities actually spend on medical research in the United Kingdom each year (figures based on previous data [3,25]). The actual levels of spending on medical research may be very different from what respondents would have been expecting. Comparing scenarios 1 and 2 allows us to test whether respondents revise their choices of allocations when they become better informed about research funding levels.

In scenario 3, respondents were asked to imagine a hypothetical situation where the government has reduced its annual spending on cancer research from £150 million (as in scenario 2) to £50 million, and has spent the £100 million difference on more research into diseases other than cancer. The crowding out hypothesis suggests that respondents would seek to make up the gap by increasing the amount they give to cancer research charities. Alternatively, respondents may view the government’s decision to redirect resources away from cancer research towards other areas of medical research as a signal that cancer research is a relatively low priority for society (hence comparing scenarios 2 and 3 allows us to test the signalling hypothesis). If so, they may follow suit by reducing the amount they give to cancer research charities.

Scenario 4 replicates scenario 3, except that the government does not increase its spending on other areas of medical research, so total annual government spending on medical research has been reduced by the same £100 million that has been cut from spending on cancer research.

Finally, in scenario 5, the government has increased its annual spending on cancer research by £100 million and has found that money by reducing its spending on research into diseases other than cancer by £100 million. It was hypothesised that, if a respondent reacts to hearing that the government has reduced its spending on cancer research by increasing (or reducing) the share of the £100 allocation they give to cancer research charities, then they may react to hearing the government has increased its spending on cancer research by reducing (or increasing) the share for cancer research charities.

Information about the levels of funding for research from charities remained constant throughout the scenarios, and was included to provide some context to the respondents. Once the respondents had completed the questions relating to the five scenarios, they were invited to provide comments to support their answers. Respondents who had claimed not to have given money to any cancer research charities in the previous year were asked what, if anything, might encourage them to donate to a cancer research charity.

The next question sought to elicit respondents’ views more directly, asking them whether hearing that the government has reduced its spending on cancer research would make them more or less likely to donate to a cancer research charity, or to donate more or less than they already do. Alternatively, respondents could indicate that government spending decisions make no difference to their donation decisions.

Finally, the respondents were asked some background questions about the types of charity donations they had made in the last year (to any kind of charity, medical or otherwise); whether the level of their charity donations had been going up, going down, or remained about the same; and whether they had any personal experience of cancer.

### Administration of survey

The questions were included in a self-completion web-based survey. The survey was administered on a sample of adult members of the United Kingdom general public, all of whom were members of a panel managed by Aurora MR, a market research agency. We sought a sample that was representative of the general population in terms of age and sex. We also sought respondents from different socioeconomic grades, choosing to oversample those from the very highest grades (A and B) in order to obtain a large subsample comprising individuals who might be expected *a priori* to be more likely than average to be regular givers of large charity donations (or to become such givers in the future). Screen-in questions, combined with a “minimum quota” approach, were used to ensure that the sample comprised individuals with the appropriate characteristics. Respondents were compensated for taking part by way of “reward points” which can be redeemed for gift vouchers.

**Figure 1:**
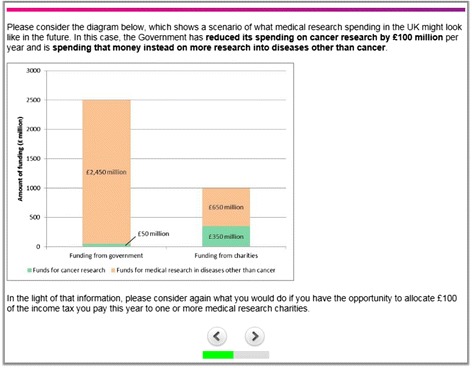
**Screenshot from web-based survey (scenario 3).**

Information about the scenarios was presented using a combination of text descriptions and diagrams (see Figure 1 for an example screenshot). All responses were recorded via the web-based survey. In order to control for potential ordering effects, the respondents were randomly assigned to one of two blocks that determined the order in which the scenarios were presented to them. Respondents in the first block faced the scenarios in the same order as described above (12345); respondents in the second block were presented with the scenario describing an increase in the government’s funding for cancer research before proceeding to the scenarios describing reductions in the government’s funding for cancer research (12534).

The study design was informed by a focus group, which was used to pilot a draft paper version of the survey and to seek feedback from general public participants. The design of the draft survey had itself been informed by feedback on several alternative designs received from two experts in stated preferences and hypothetical survey design. The focus group took place in London in May 2013. Nine members of the general public took part, all of whom claimed to support the principle of giving to charity.

The comments received were largely favourable, with participants describing the scenarios as interesting and easy to understand, and claiming that the survey was straightforward to complete without assistance. However, the participants also criticised the survey for being overly “wordy” and repetitive, and some participants questioned the plausibility of some of the scenarios.

The findings from the focus group informed the design of the final survey in a number of ways, in particular, reduced repetition from scenario to scenario; the use of diagrams (rather than tables) to demonstrate the key pieces of information in the scenarios; and the use of features to make the web-based survey more user-friendly than its pen-and-paper counterpart. We also made the reference to the Italian tax hypothecation policy more prominent, as participants felt that knowing this made the hypothetical scenarios seem more realistic.

### Ethics

Ethical review was not required for this study, in accordance with the National Health Service National Research Ethics Service algorithm for determining whether review by an National Health Service Research Ethics Committee is required [25].

## Results

### Overview of the sample

The survey was carried out in the United Kingdom in July 2013. Respondents who completed the survey in less than 3.5 minutes were excluded from the sample due to concerns about data quality (n = 74), leaving a sample of 401 respondents. Table 2 presents the background characteristics of the sample. By design, the sample was broadly representative of the general United Kingdom population with respect to age and sex [26], and comprised a larger proportion of individuals in the highest socioeconomic grades [27].

**Table 2:** **Sample background characteristics**

	**Freq**	**%**
**Total**	**401**	**100**
Sex		
Male	214	53.4
Female	187	46.6
Age (years)		
18–29	49	12.2
30–39	90	22.4
40–49	63	15.7
50–59	76	19.0
60 and over	123	30.7
Social grade (refers to the occupation/responsibilities of the chief wage earner of the respondent’s household)		
A (higher managerial, administrative, or professional)	23	5.7
B (intermediate managerial, administrative, or professional)	144	35.9
C1 (supervisory or clerical and junior managerial, administrative, or professional)	95	23.7
C2 (skilled manual workers)	52	13.0
D (semi-skilled and unskilled manual workers)	27	6.7
E (state pensioners, casual and lowest grade workers, unemployed with state benefits only)	60	15.0
Types of charity donations made in the last year		
Money – regular donation	127	31.7
Money – one-off donation	241	60.1
Money – other (charity events, auctions, etc.)	126	31.4
Non-financial (donation of unwanted goods, volunteering, etc.)	223	55.6
None of the above	45	11.2
Over the last 3 years, what has happened to the level of your charity donations(s)?		
Gone up	66	16.5
Gone down	56	14.0
About the same	279	69.6
Personal experience of cancer (respondents could tick multiple boxes)		
Yes, self	34	8.5
Yes, close friend or relative	275	68.6
No	87	21.7
No answer given	22	5.5

### Respondents’ guesses about government spending levels

**Figure 2:**
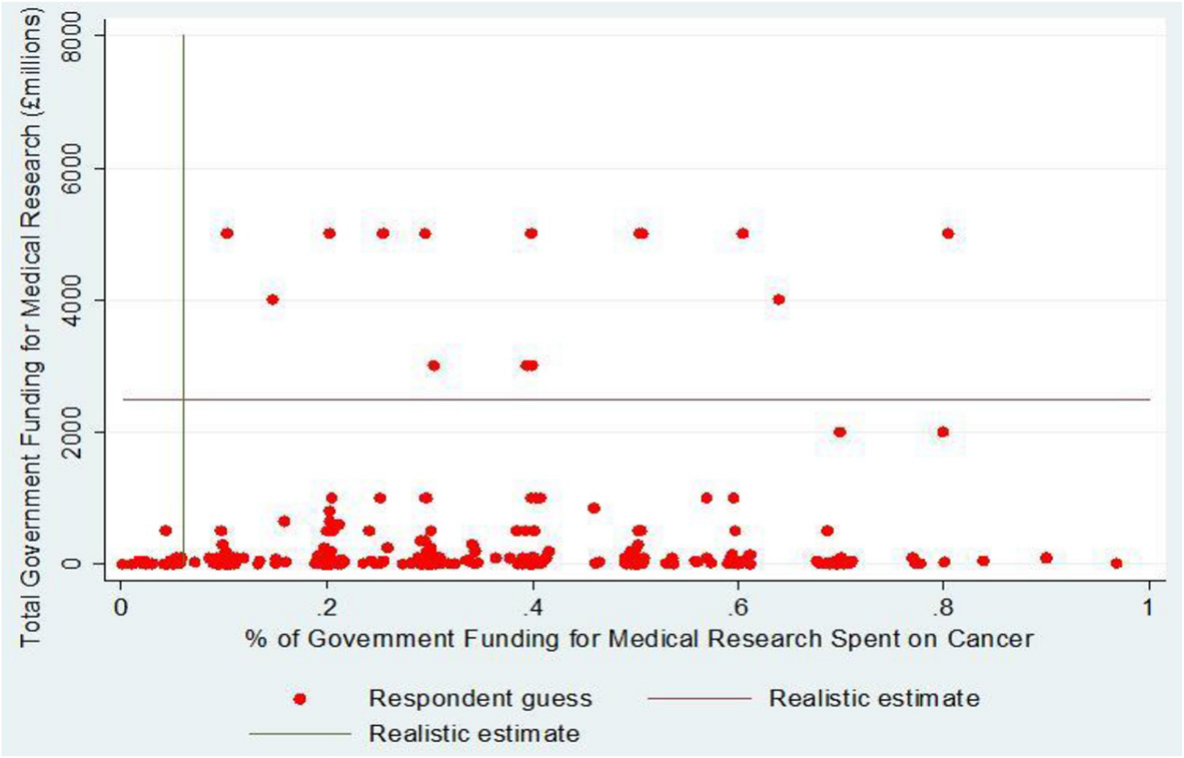
**Distribution of guesses of levels of government spending.**

Figure 2 shows the distribution of answers given by respondents when asked: (1) how much the United Kingdom government spends on medical research each year, in millions of pounds (vertical axis) and (2) what proportion of the total United Kingdom government spending on medical research each year is spent on cancer research (horizontal axis). The vast majority (96.3%) of respondents underestimated total government spending on medical research (median guess = £24 million; actual figure approx. £2,500 million), and a similarly large majority overestimated the proportion of government spending on medical research that is spent on cancer research (median guess approx. 30%; actual figure approx. 6%; actual figures based on [3,28]). The majority of respondents guessed that government funding of cancer research had either been going down (33.7%) or remained about the same (35.2%) over the past 3 years, with most of the remainder selecting the “don’t know” option.

### Responses to the scenario questions

Table 3 reports the aggregate response data for the five scenarios. In scenario 1, in which no information about actual levels of medical research funding was provided, the respondents were fairly equally split between giving the majority of the £100 allocation of their income tax payment to cancer research charities (38.2%), giving the majority to non-cancer medical research charities (27.9%), and splitting the allocation equally between cancer research and non-cancer medical research charities (33.9%). In all of the subsequent scenarios, the proportion of respondents giving the majority of the allocation to cancer research charities was greater than in scenario 1. Respondents were most likely to give the majority of the allocation to cancer research charities in scenarios 3 and 4, when they were asked to imagine that the government had reduced its spending on cancer research. One hundred and fifty-three respondents (38.2%) opted for the same split between cancer research and other medical research charities in all five scenarios, including 48 respondents (12.0%) who chose a 50:50 split on every occasion.

**Table 3:** **Aggregate response data for scenarios 1 to 5**

	**Scenario**				
	**1**	**2**	**3**	**4**	**5**
	**No info**	**Realistic estimates**	**↓ Cancer research; ↑ other medical research**	**↓ Cancer research**	**↑ Cancer research; ↓ other medical research**
Allocation to cancer research charities (out of notional £100 tax deducted sum)					
<£50	27.9%	27.9%	26.9%	25.7%	29.4%
£50	33.9%	22.2%	16.5%	19.7%	25.4%
>£50	38.2%	49.9%	56.6%	54.6%	45.1%
Mean allocation to cancer research charities	£53.60	£58.19	£63.15	£62.35	£56.47
Change in personal out-of-pocket donations to cancer research charities^a^					
Would reduce	4.0%	1.8%	2.6%	2.2%	5.8%
Would increase	4.7%	9.1%	11.7%	9.5%	5.1%
Would not change	91.2%	89.1%	85.8%	88.3%	89.1%
Mean change in personal out-of-pocket donation to cancer research charities^b^	–£0.10	+£1.12	+£1.54	+£1.51	–£0.21
Change in personal out-of-pocket donations to other medical research charities^c^					
Would reduce	5.4%	8.3%	7.8%	5.4%	4.4%
Would increase	2.5%	2.0%	3.9%	3.9%	5.4%
Would not change	92.2%	89.7%	88.2%	90.7%	90.2%
Mean change in personal out-of-pocket donations to other medical research charities	–£0.52	–£0.78	–£0.37	+£0.09	+£0.02

^a^Questions asked only to respondents who had given money to one or more cancer research charities in the previous year

^b^Excludes outlier (individual who claimed that they would increase their personal donations by £250)

^c^Questions asked only to respondents who had given money to one or more non-cancer medical research charities in the previous year.

↓ denotes a reduction in government spending

↑ denotes an increase in government spending

On average, from the £100 of income tax they were able to allocate to medical research, respondents chose to give £58.19 to cancer research charities and £41.81 to other medical research charities in scenario 2, in which they were presented with realistic estimates of government spending levels. In the scenarios in which the government had *reduced* its spending on cancer research, the average amount from within the £100 of income tax to be allocated to medical research that would be given to cancer research charities *increased* by £4.96 and £4.16 (scenarios 3 and 4, respectively). In the scenario in which the government had *increased* its spending on cancer research, the average amount given to cancer research charities from within the £100 of income tax to be allocated *fell* by £1.72 per person, on average (scenario 5). The mean allocation to cancer research charities in scenario 2 is statistically significantly smaller than the corresponding mean allocations in scenarios 3 (*t*-test; *P* <0.01) and 4 (*P* <0.05). However, it is not statistically significantly greater than the corresponding mean allocation in scenario 5 (*P* >0.05).

In all five scenarios, of the respondents who had earlier claimed to have given money to medical research charities in the previous year, the vast majority (ranging from 88.2% scenario 3 to 92.2% in scenario 1) said that they would not change the level of their personal out-of-pocket charity donations even after being given the opportunity to give an extra £100 out of their income tax. These respondents were most likely to increase their out-of-pocket donations to cancer research charities in scenarios 3 and 4, when they were asked to assume that the government had reduced its spending on cancer research; and were most likely to increase their out-of-pocket donations to non-cancer medical research charities in scenario 5, when they were asked to assume that the government had increased its spending on cancer research and had found that money by reducing its spending on research into diseases other than cancer.

Overall, 274 out of the 401 respondents (68.3%) said that they had donated to cancer research charities in the last year. Among that group of respondents, Table 3 shows that the mean personal out-of-pocket donation to cancer research charities would be greater in scenario 4 than in scenario 2, but by just £0.39 per existing donor. We do not observe a statistically significant difference in the mean change in personal out-of-pocket donations when comparing scenario 2 with scenarios 3, 4, or 5 (*t-*test; *P* >0.05 in all three cases). However, the mean change in personal out-of-pocket donation is statistically significantly smaller in scenario 5 than in scenario 3 (*P* <0.05).

**Figure 3:**
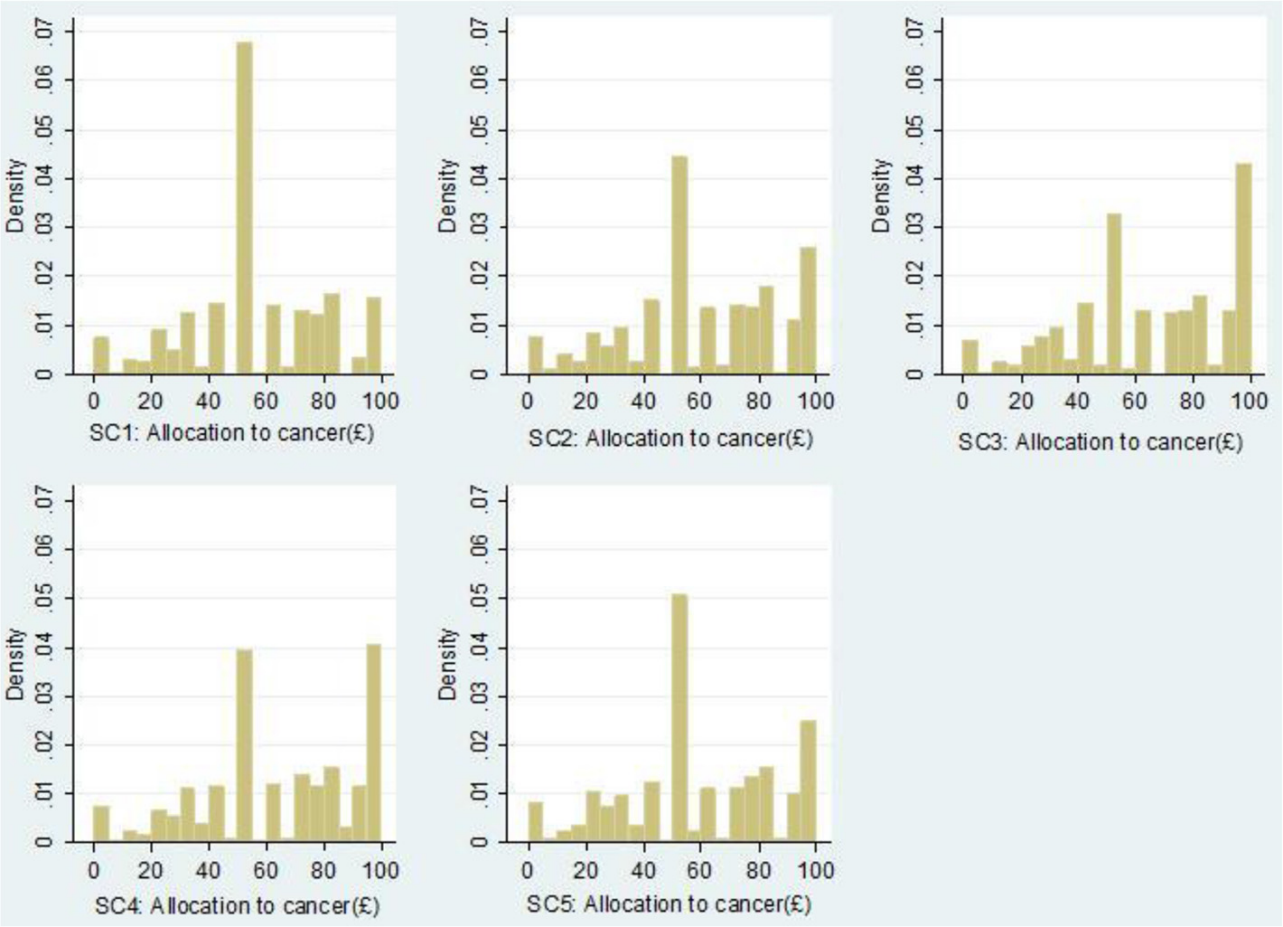
**Distributions of the allocations given to cancer research charities.**

Figure 3 shows the distributions of the allocations given to cancer research charities in each of the five scenarios. The tendency to choose an even split between cancer research and other medical research charities is greatest in scenario 1. After being provided with information about the levels of government spending on medical research (scenarios 2 to 5), some respondents switch to giving the entire allocation to cancer research charities. This tendency is strongest in the scenarios that describe cuts in government spending on cancer research. It is notable that the distributions for scenarios 2 and 5 are near-identical.

Across all scenarios, respondents with personal experience of cancer were less likely to choose even splits than those without personal experience of cancer. The association between having personal experience of cancer and the propensity to choose an even split is statistically significant (χ^2^ test; *P* <0.01). Respondents with personal experience of cancer gave larger allocations to cancer research charities on average – the difference in mean allocations between the experience/no experience groups was statistically significant (*t*-test; *P* <0.01). The differences in the distributions of the allocations between these groups were statistically significant in scenarios 3 and 4 (Kolmogorov-Smirnov test), where respondents with personal experience of cancer were more likely than those without personal experience of cancer to give a larger share of the allocation to cancer research. No statistically significant differences were found between the allocations of respondents in the highest socioeconomic grades and those of respondents in the lower grades.

**Figure 4:**
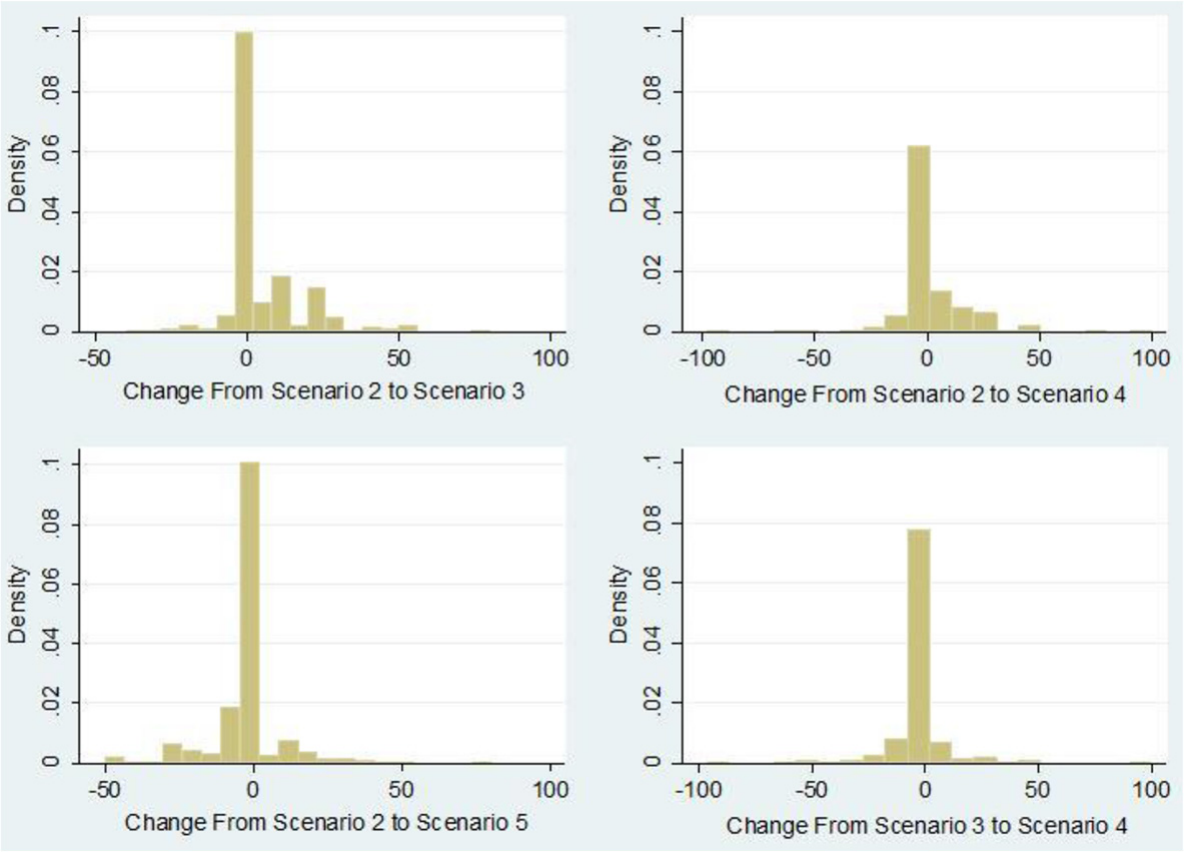
**Changes in allocations from one scenario to another (for selected pairs of scenarios).**

Figure 4 shows the extent to which respondents changed their allocation choices from one scenario to another (for selected pairs of scenarios). Compared to scenario 2 (the scenario presenting realistic government spending estimates), respondents were more likely to give an increased share of the allocation to cancer research charities in scenarios 3 and 4 (the scenarios describing cuts to government funding for cancer research), and were slightly more likely to give a reduced share in scenario 5 (the scenario describing an increase in government funding for cancer research).

Comparisons of respondents’ preferred allocations of the £100 of their income tax with their subsequent stated changes in personal out-of-pocket donations to cancer and non-cancer medical research charities indicated the following: the larger a respondent’s allocation to cancer research charities, the more likely that respondent is to increase (or reduce) their personal out-of-pocket donations to cancer research (or non-cancer medical research) charities as a result of being given the opportunity to allocate some of their income tax. The relationship between respondents’ preferred allocations and the changes in their personal donations to cancer research charities appears to be strongest in scenarios 3 and 4.

In order to identify the drivers of choices whilst taking into account clustering at the respondent level, we used the STATA 13.1 software to estimate a random effects model using a maximum-likelihood estimator. This model takes account of the fact that multiple responses are obtained from the same individual and could be correlated. The dependent variable corresponded to the allocation given to cancer research charities. The statistical significance of a number of independent variables was tested. These covered background characteristics (e.g. sex, prior donations in the last year), the order in which the scenarios were presented (scenario 5 prior to scenarios 3 and 4, or vice versa), dummies for each scenario or “treatment effect” (with scenario 1 assigned as the base scenario), and selected interactions (e.g. order of the scenarios interacted with treatment effects).

Model performance was assessed by examining the Akaike and Bayesian information criteria; the base model included only the dummies for each scenario. This model was compared with a group of models that each included the scenario dummies plus one of the independent variables. The model with the lowest Akaike and Bayesian information criteria was selected. This was in turn compared with a group of models that added a further independent variable out of those remaining. The process was repeated until adding further independent variables did not improve Akaike and Bayesian information criteria. The results of the best-fitting model are shown in Table 4.

**Table 4:** **Random effects model results** ^**a**^

	**Best-fitting model (all respondents)**			**Best-fitting model (sub-sample randomised to ordering 12345)**			**Best-fitting model (sub-sample randomised to ordering 12534)**			**Best-fitting model (all respondents) plus variable for ordering**		
	**Coefficient**		**Std. Err.**	**Coefficient**		**Std. Err.**	**Coefficient**		**Std. Err.**	**Coefficient**		**Std. Err.**
Constant	47.39	***	4.54	50.94	***	6.76	43.87	***	6.21	43.38	***	4.70
Treatment effects (dummy variables)												
Scenario 2	4.59	***	0.78	5.05	***	1.09	4.14	***	1.12	4.59	***	0.78
Scenario 3	9.55	***	0.78	10.47	***	1.09	8.64	***	1.12	9.54	***	0.78
Scenario 4	8.75	***	0.78	9.86	***	1.09	7.66	***	1.12	8.75	***	0.78
Scenario 5	2.87	***	0.78	2.94	***	1.09	2.79	**	1.12	2.87	***	0.78
Personal experience of cancer (YES = 1)	5.69	**	2.81	4.71		4.29	6.29	*	3.74	5.53	**	2.82
Personal donation to Cancer Research UK in the last year (YES = 1)	10.38	***	2.57	9.37	**	3.75	11.28	***	3.56	10.34	***	2.57
Personal donation (any type) in the last year (YES = 1)	−7.58	*	4.11	−12.28	**	5.77	−1.81		5.89	−7.42	*	4.11
Guess of percentage of government spending on medical research that is on cancer research	9.95		6.33	17.27	*	9.06	0.64		8.91	9.94		6.32
Order in which scenarios were presented (12345 = 0; 12534 = 1)	Not included			Not included			Not included			2.06		2.46
Number of observations (panel data)	2005			990			1015			2005		
Number of respondents	401			198			203			401		
Likelihood-ratio test – χ^2^	219.85	***		136.10	***		92.10	***		220.55	***	

Significant to the: ***=1% level; **=5% level; *=10% level.

^a^Further details of the regression and the results of other variables tested are available on request.

The coefficients for the dummies for scenarios 2 to 5 (in relation to scenario 1) were all positive and statistically significant. The dummy for respondents’ personal experience of cancer was also positive and statistically significant, which indicates that respondents with personal experience of cancer were more likely to give larger allocations to cancer research charities than those without that experience. However, none of the coefficients for the interactions between personal experience and the scenario dummies were statistically significant, which suggests that, although personal experience was a driver of respondents’ overall allocation choices, it did not affect the way in which their allocation choices were influenced by information about government spending decisions in the various scenarios. The order in which the scenarios were presented was not found to affect respondents’ overall allocations.

We used the Wald test and pairwise comparisons of marginal predictions to compare the overall allocations for all combinations of scenarios. We found that the differences between scenarios were statistically significant at the 5% level in all cases with the exception of the difference between scenarios 3 and 4 (both of which described a reduction in spending on cancer research).

### Responses to the direct attitudinal question

When asked directly whether, if they were to hear that the government had reduced its spending on cancer research, that would make them more or less likely to donate to a cancer research charity (or to donate more or less than they already do), the majority of respondents (70.8%) claimed that government spending decisions make no difference to their decision about whether or not (or how much) to donate to a cancer research charity. Of the remainder, most claimed that it would make them more likely to donate, whereas only eight respondents (2.0%) claimed that it would make them less likely to donate. Table 5 provides a cross-tabulation of respondents’ answers to this question and their answers to the question regarding scenario 4 when they were asked if they would change the level of their personal out-of-pocket donations to cancer research charities when faced with a situation whereby the government had reduced its spending on cancer research by £100 million (the wording of this scenario most closely matches the wording of the direct attitudinal question). Note that Table 5 refers only to the 274 respondents who had given money to cancer research charities in the previous year.

**Table 5:** **Cross tabulation – question regarding scenario 4 about impact on personal out-of-pocket donations versus direct attitudinal question**

		**Direct attitudinal question**			
		**Less likely to donate**	**Makes no difference**	**More likely to donate**	**Total**
Question regarding scenario 4 (impact on out-of-pocket donations)	Would reduce	0	2	4	6
	Would not change	6	171	65	242
	Would increase	1	8	17	26
Total		7	181	86	274

### Qualitative data – comments made by respondents

All respondents were invited to leave comments about their answers to scenario questions, 78 of whom (19.5%) opted to do so. The comments were then coded and organised into categories. The most common category of comment (made by 12 respondents) referred to the notion that the respondent’s personal donations would not be affected by changes in government policy (for example, “I would still pay the same amount regardless of how much the government funds as it is private to me how I want to spend money”). Other common categories of comment included surprise/disgust at actual government funding levels and explanations by respondents that they tend to give money to research focused on disease areas that they are connected to in some way (both made by nine respondents each).

Respondents who had not earlier claimed to have given money to cancer research charities in the previous year were asked what might encourage them to do so; 72 of the 127 respondents who were asked this question (56.7%) left a comment of some sort. Again, the comments were coded and organised into categories. The most common factors suggested were personal experience of cancer (mentioned by 15 respondents, predominantly those in the higher) and having a larger disposable income (mentioned by 13 respondents, predominantly those in the lower socioeconomic grades). None of the respondents who were asked this question gave a response that mentioned the level of government funding for cancer (or any other type of) research.

## Discussion

This study has elicited the views of a sample of the United Kingdom general public about how (hypothetical) changes in government spending on cancer research might affect their own willingness to donate to cancer research charities, and to hypothecate some of their income tax to them. We found that most people’s tax hypothecation and private donation decisions to cancer research charities were only slightly affected by information about government spending on cancer research. When respondents were asked to suppose that the government had cut funding for cancer research, the overall tendency was to give a slightly larger share to cancer research charities of the £100 of their income tax that they were told they may allocate. When respondents were asked to suppose that the government had increased funding for cancer research, the overall tendency was to give a slightly smaller share to the allocation to cancer research charities. The impact of an increase in government funding was smaller in magnitude than that of a cut. Notwithstanding these overall tendencies, most respondents’ preferred allocation splits did not vary much from scenario to scenario, and a sizeable minority (38.2%) of respondents chose the same allocation split in all five scenarios, i.e. regardless of government spending on cancer and other medical research.

In all five scenarios, the vast majority (88.2–92.2%) of respondents said that they would not take the opportunity to change the levels of their existing personal out-of-pocket donations to cancer research and/or other medical research charities, despite the fact that these charities would be receiving additional funding by way of the £100 allocation of the respondent’s income tax. For those respondents who said that they would change their personal donations, the larger their preferred allocation to cancer research charities, the more likely they were to increase their personal donations to cancer research charities.

Our finding that most people’s private donation decisions to medical research charities are only slightly affected by information about government spending on medical research is further supported by the responses to the direct attitudinal question. Approximately two-thirds of respondents claimed that hearing that the government had reduced its spending on cancer research would not affect their decision about whether to donate to a cancer research charity, or the size of their donation.

The open-ended comments made by respondents paint a similar picture. Few of the comments mentioned government funding for medical research as a factor affecting their decisions, with respondents claiming that personal experience of cancer, or increases in their disposable income, would be the main drivers behind any future decision to donate to a cancer research charity. Of the respondents who left a comment about their answers to the scenario questions, 15.4% took the opportunity to reiterate the fact their personal donations would not be affected by changes in government policy or funding levels. Nevertheless, there were some respondents whose preferred allocations varied substantially from scenario to scenario. Overall, the results suggest that crowding out effects outweigh any possible crowding in or signalling effects. Of the respondents who amended their allocation splits upon being given new information about government spending levels, the majority tended to move in the opposite direction to the government – increasing the share for cancer research charities when government funding for cancer research was cut, and reducing the share for cancer research charities when government funding for cancer research was increased.

On average, when moving from scenario 2 (realistic estimates of government spending) to scenario 4 (£100 million cut in government funding for cancer research with no increase in government funding for other medical research), respondents increased the share of the £100 of income tax allocated to cancer research by £4.16. Given that the adult population of the United Kingdom is approximately 50 million [29] and assuming that the wider population would behave in accordance with the stated preferences elicited in this study, then, if the government were to cut funding for cancer research by £100 million and gave each individual £100 of income tax to allocate to cancer research or other medical research charities of their choosing, then cancer research charities could, between them, receive £208 million from such a policy. In other words, the general public may rebalance tax spending back to cancer research, given the chance, if the government were to cut its planned spending on cancer research and to introduce a mechanism for redistributing income tax to medical research charities. The direction of intent is clear. However, not much can be read into the magnitude of the additional allocation to cancer research owing to the hypothetical nature of the exercise and the likelihood that the magnitude will be strongly affected by the size of the amount that the individual is given the discretion to allocate. In the United Kingdom, it is not currently possible to reallocate tax income in this way.

In addition, since the mean change in personal out-of-pocket donation to cancer research charities is greater in scenario 4 than in scenario 2, cancer research charities might also expect to receive a small amount of further donations from the pockets of individuals who already give money to these types of organisations, should the government cut its spending on cancer research. Comparing scenario 4 with scenario 2, the additional £0.39 per person saying they already donate to cancer research charities, who make up 68.3% of our survey respondents (274/401), suggests that, if replicated across 68.3% of the 50 million United Kingdom adult population, this would amount to extra out-of-pocket donations of £13 million. This would not go far towards offsetting the hypothetical £100 million cut in government spending on cancer research.

However, we would urge caution when scaling up in this way: in reality, even the better-informed members of the public would be unlikely to have access to information about government spending levels as presented in the scenarios. Furthermore, means tend to be skewed by extreme values (such as respondents who claim that they would give an additional £100 to cancer research charities after already having been given the opportunity to allocate £100 of income tax to those charities) that may not accurately reflect what would happen if the scenario were actually to occur. It is perhaps more telling that the median change in personal out-of-pocket donations to both cancer research and other medical research charities was zero in all five scenarios.

It is not surprising that the respondents were largely uninformed about current levels of government spending on medical research. A survey of public views about science and biomedical research reported that, when asked which groups they were aware of that carry out medical research in the United Kingdom, only 6% mentioned the government, 18% mentioned the National Health Service, 23% mentioned universities, 48% mentioned medical research charities, and 16% said that they did not know [30].

Most respondents guessed that government spending on medical research is far more concentrated on cancer research than is actually the case. This may explain the large shares of the allocations given to cancer research at the expense of other medical research (in each of the scenarios, the mean amount given to cancer research charities was greater than £50 of the £100 to be allocated). However, the fact that cancer research was clearly the main subject of the survey, and the fact that respondents were informed that the study was funded by Cancer Research UK, is likely to have resulted in a focusing effect whereby respondents placed more importance on cancer than they otherwise might have done. In terms of the purpose of this study, however, the actual amounts given to cancer research charities in any given scenario are less important than the ways in which those amounts change from scenario to scenario.

After having been given the opportunity to allocate £100 of income tax to medical research charities of their choosing, very few respondents then said that they would take the opportunity to reduce the size of their existing personal out-of-pocket donations. Most existing donors to medical research charities said that they would not change the size of their personal donations. This is particularly the case in scenario 1, in which respondents were not given any information about government funding levels. This means that their answers regarding personal donations under scenario 1 would not have been driven by concerns that the government is spending too little (or too much) on medical research.

However, a drawback of stated preference studies is that they only elicit data on what respondents say that they would do/prefer – we do not know whether they would behave in the same way if the hypothetical scenarios were actually to happen. Survey respondents may exaggerate claims about their positive behaviour (i.e. giving to charity) either to appease or impress the researcher, or because they have a deluded view of themselves. Future research could combine the stated preference design with an experimental lab-based study in order to test whether people act on their claims when given real money to allocate.

Another limitation of such studies, particularly those administered over the Internet without an interviewer on hand to provide guidance, is that it is difficult to know whether respondents paid sufficient attention to the tasks and took the scenarios seriously. We therefore excluded respondents who completed the survey implausibly quickly (in less than 3.5 minutes). We also tested the impact of applying alternative (stricter) cut-offs; these did not affect our general findings.

A further issue is the potential for experimenter demand bias, whereby respondents give responses that they believe the researchers are expecting. However, given the competing theories of crowding out and crowding in, we had no *a priori* expectations about the direction of impact of providing information about cuts to or increases in government funding levels. Furthermore, even if the study design had led respondents to infer that they were expected to change their allocations and out-of-pocket donations in response to changes in the information provided, we still observe few instances of respondents doing so.

## Conclusions

The results of our stated preference survey of 401 adults in the United Kingdom lead to the overall conclusion that the public’s decisions about how much to donate to cancer research or other medical research charities are not greatly affected by changes to government plans about the amount of public funding of cancer or other medical research. Personal experience of cancer appears to be a more important driver of people’s decisions to donate to cancer research charities.

## Endnote

^a^ Though a large proportion of this funding is provided by charities that do not fundraise from the general public.
