# Erratum to: Government and charity funding of cancer research: public preferences and choices

**DOI:** 10.1186/s12961-015-0030-y

**Published:** 2015-10-06

**Authors:** Koonal Kirit Shah, Jon Sussex, Karla Hernandez-Villafuerte

**Affiliations:** Office of Health Economics, Southside 7th floor, 105 Victoria Street, London, SW1E 6QT UK

## Erratum

After publication of the original article [1] it came to the publisher’s attention that reference citations 26, 27 and 28 and the corresponding entries in the References list were incorrect. These errors have now been corrected in the original article.
